# Novel Sources of Pre-Harvest Sprouting Resistance for Japonica Rice Improvement

**DOI:** 10.3390/plants10081709

**Published:** 2021-08-19

**Authors:** Jae-Sung Lee, Dmytro Chebotarov, Kenneth L. McNally, Valerien Pede, Tri Deri Setiyono, Rency Raquid, Woong-Jo Hyun, Ji-Ung Jeung, Ajay Kohli, Youngjun Mo

**Affiliations:** 1 International Rice Research Institute, Los Baños 4031, Philippines; js.lee@irri.org (J.-S.L.); d.chebotarov@irri.org (D.C.); K.McNally@irri.org (K.L.M.); v.pede@irri.org (V.P.); Tsetiyono@agcenter.lsu.edu (T.D.S.); r.raquid@irri.org (R.R.); 2 National Institute of Crop Science, Rural Development Administration, Wanju 55365, Korea; onlybio@korea.kr (W.-J.H.); jrnj@korea.kr (J.-U.J.); 3 Department of Crop Science and Biotechnology, Jeonbuk National University, Jeonju 54896, Korea

**Keywords:** pre-harvest sprouting, japonica rice, plant hormones, ABA, GA, GWAS

## Abstract

Pre-harvest sprouting (PHS), induced by unexpected weather events, such as typhoons, at the late seed maturity stage, is becoming a serious threat to rice production, especially in the state of California, USA, Japan, and the Republic of Korea, where japonica varieties (mostly susceptible to PHS) are mainly cultivated. A projected economic loss by severe PHS in these three countries could range between 8–10 billion USD per year during the next 10 years. Here, we present promising rice germplasm with strong resistance to PHS that were selected from a diverse rice panel of accessions held in the International Rice Genebank (IRG) at the International Rice Research Institute (IRRI). To induce PHS, three panicle samples per accession were harvested at 20 and 30 days after flowering (DAF), respectively, and incubated at 100% relative humidity (RH), 30 °C in a growth chamber for 15 days. A genome-wide association (GWA) analysis using a 4.8 million single nucleotide polymorphisms (SNP) marker set was performed to identify loci and candidate genes conferring PHS resistance. Interestingly, two tropical japonica and four temperate japonica accessions showed outstanding PHS resistance as compared to tolerant indica accessions. Two major loci on chromosomes 1 and 4 were associated with PHS resistance. A priori candidate genes interactions with rice gene networks, which are based on the gene ontology (GO), co-expression, and other evidence, suggested that a key resistance mechanism is related to abscisic acid (ABA), gibberellic acid (GA), and auxin mediated signaling pathways.

## 1. Introduction

Pre-harvest sprouting (PHS), the germination of seeds on mother plants before harvest, occurs in many cereal crops, such as wheat, barley, and rice, when seeds lose dormancy under warm and humid circumstances prior to harvest [1,2]. Seed dormancy is a critical trait for survival in nature because it suppresses germination until favorable environmental conditions occur for plant growth. However, as domestication has imposed selection pressure against strong dormancy so seeds can be sown right after harvest and emerge simultaneously for uniformity, most modern crop varieties have much weaker dormancy than their wild ancestors [3]. The excessive loss of dormancy can cause serious PHS damage, decreasing crop yield and resulting in significant economic loss [4]. In addition to yield reduction, PHS deteriorates the eating, cooking, and processing qualities by altering starch physicochemical properties, such as reduced amylose and short-chain amylopectin content, and irregular starch granule structures [5,6]. As compared to wheat or barley, PHS has been much less of a threat to rice production since the weather during rice harvest seasons is relatively dry and cool. However, recent climate change has caused unexpected rains and typhoons during seed maturity periods that have affected rice production in China and the Republic of Korea [7,8]. Therefore, improving popular rice varieties for PHS resistance should be considered as an essential breeding target.

Genetic factors conferring PHS resistance have been shown to involve flavonoid biosynthesis and ABA signaling pathways [2]. The association between seed pericarp color and PHS resistance has been known for a long time, with red wheat generally having stronger dormancy than white wheat [9]. *Tamyb10* genes on the long arms of group 3 chromosomes underlie the *R-1* loci for seed pericarp color in wheat and encode Myb-type transcription factors involved in flavonoid biosynthesis [10,11]. In the case of rice, the map-based cloning of *qSD7-1*/*qPC1* identified the *Rc* gene, encoding a basic helix-loop-helix family transcription factor, which upregulates genes involved in the biosynthesis of both flavonoid and ABA [12]. In the T-DNA/Tos17 insertional mutant populations, genes involved in the biosynthesis of carotenoids (i.e., *OsPDS*, *OsZDS*, *OsCRTISO*, and β-*OsLCY*), the important precursors of ABA, were identified [13]. A similar study using rice mutants with the PHS phenotype identified causal mutations in *OsCNX1* and *OsCNX6*, the genes encoding molybdenum cofactors required for ABA biosynthesis [14]. Also, characterization of *phs9-D* revealed that this PHS mutant in rice carries a dominant mutation in *PHS9*, a gene encoding a CC-type glutaredoxin, which mediates ABA signaling through the interaction with ABA receptors [15]. In spite of these findings underlying clear biochemical mechanisms on PHS resistance, breeding efforts enhancing PHS resistance in rice varieties have been limited due to lack of genetic resources. *Sdr4* and *qSD7-1*/*qPC1*, the major loci associated with PHS resistance were from a single genetic resource: Aus landrace Kasalath having red pericarp color and strong dormancy [12,16]. The aims of this study were to (1) screen a panel of japonica rice accessions for PHS resistance; and (2) identify japonica specific-loci conferring the resistance, which could be effectively used for improving popular japonica varieties highly susceptible to PHS.

## 2. Results

### 2.1. Optimal Sampling Time for PHS Screening

**Figure 1:**
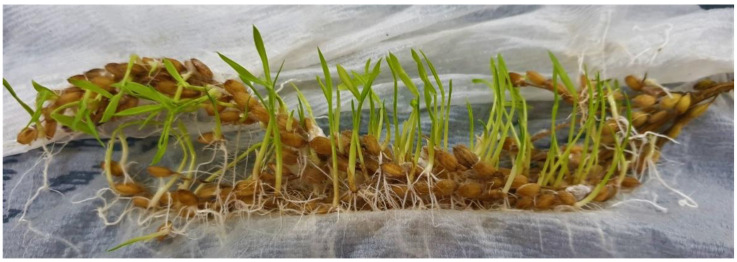
Pre-harvest sprouting of temperate japonica rice harvested at 20 days after flowering on the Zeigler Experiment Station of the International Rice Research Institute, Philippines, in the 2019 dry season.

**Figure 2:**
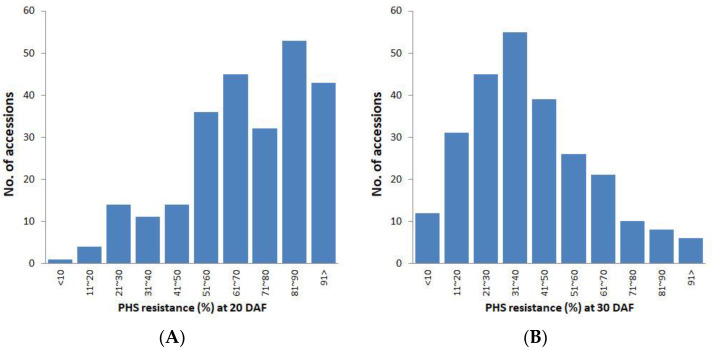
Comparison of pre-harvest sprouting resistance of the temperate and tropical japonica panels, between samples harvested at 20 (**A**) and 30 (**B**) days after flowering.

Although PHS was observed between early and late seed maturity stages at the IRRI station (Figure 1), it had not been defined for when PHS stress level is highly likely to increase under Philippine weather conditions. To optimize sampling time for PHS screening, we collected panicle samples at early–mid (20 DAF) and late (30 DAF) seed maturity stages, respectively, and compared the level of resistance between the two groups (Figure 2). In general, PHS resistance was higher in the 20 DAF group with a mean of 69.08% than 30 DAF group with a mean of 40.67%. However, resistance in many accessions reduced between 20 and 30 DAF. Especially, 48 accessions with high resistance above 90.00% at 20 DAF significantly lost their resistance at 30 DAF (mean of reduced resistance: 34.59%). Data of the 30 DAF group were therefore more suitable for both selection of resistant germplasm and genetic analysis.

### 2.2. Intraspecific Variation on PHS

**Figure 3:**
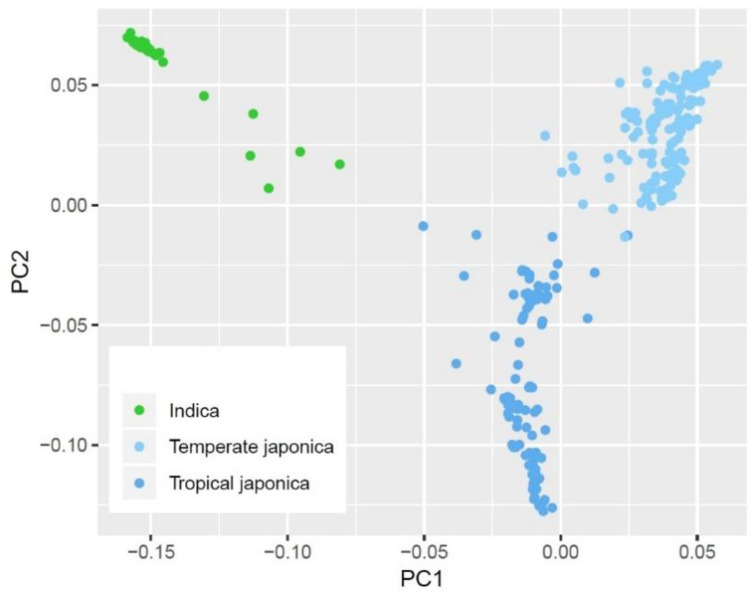
Principal component analysis showing genetic variation between indica (green), temperate japonica (light blue), and tropical japonica (dark blue).

**Figure 4:**
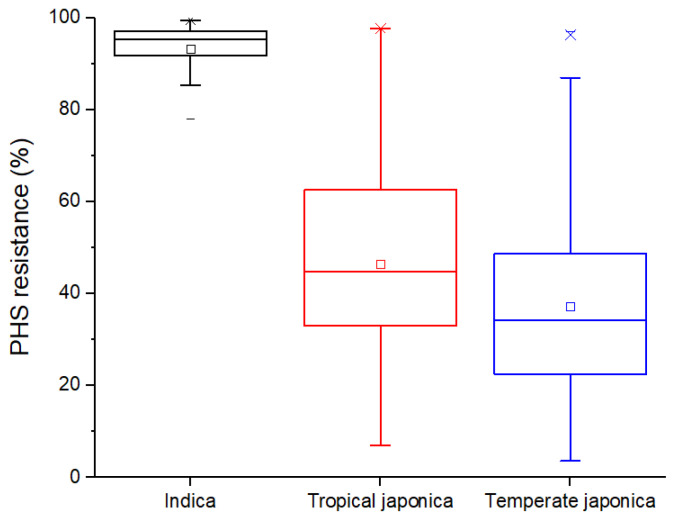
Pre-harvest sprouting resistance variation of indica (**black**), tropical japonica (**red**), and temperate japonica (**blue**) rice sub-groups harvested at 30 days after flowering.

To assess population structure in our dataset, we performed principal components (PC) analysis, which confirmed an intraspecific genomic variation among three rice sub-groups used in this study (Figure 3), consistent with earlier studies. The first PC clearly separated the indica group from the two japonica groups, while the second PC separated temperate japonica and tropical japonica groups with some overlapping regions in the middle. As compared with japonica groups, PHS resistance within the indica group was very high with relatively small variation in the range of 78.08% and 99.50% (mean: 93.33%) (Figure 4). On the other hand, PHS resistance varied largely within each japonica group; it ranged between 7.01 and 97.63% (mean: 46.36%) in tropical japonica and 3.53% and 96.89% (mean: 37.13%) in the temperate japonica group. Interestingly, two tropical japonica accessions from Bhutan and Guinea-Bissau and four temperate japonica accessions from Cambodia, Georgia, Nepal, and the United States recorded outstanding PHS resistances above 90.00%.

### 2.3. Loci Associated with PHS Resistance

**Figure 5:**
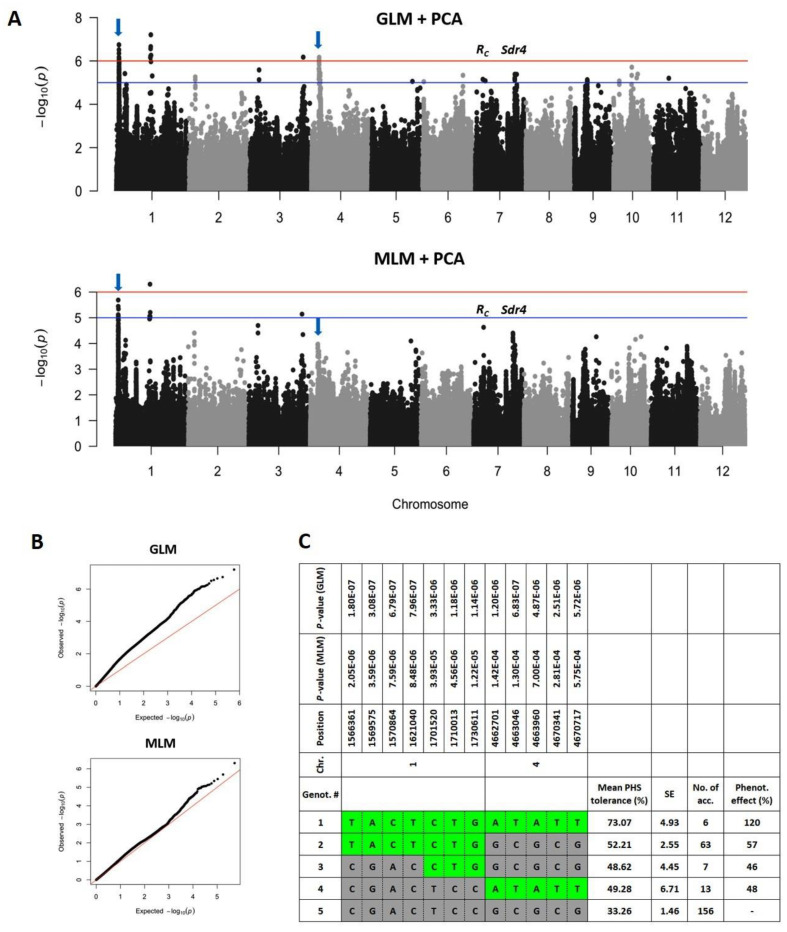
Genome-wide association analysis of pre-harvest sprouting (PHS) of Japonica rice accessions: (**A**) Manhattan plot generated through the general linear model (GLM) with principal components and mixed linear model (MLM) with kinship matrix and principal components. Points above the blue and red threshold lines indicate significant association at −log10(*p*) = 5 and 6, respectively. Arrows indicate two major loci used for haplotype analysis in Figure 5C. *R_C_* and *Sdr4* loci are major dormancy genes in rice; (**B**) quantile–quantile plots; and (**C**) haplotype effects on the phenotype trait.

An initial GWA analysis using the whole data set detected a number of loci statistically associated with PHS resistance (Supplementary Material S1). Since 24 indica accessions having strong PHS resistance comprised a small portion (8.7%) of the panel and they genetically differed from japonica groups (Figure 3), we also conducted GWA analysis excluding indica data in order to diminish the confounding effect due to subpopulation structure. As a result, most loci above the significance threshold did not attain significance in a GWA model including japonica groups only (Figure 5A). This perhaps indicates that initial analysis might reflect a large genomic variation between indica and japonica groups rather than PHS resistance per se. Independent studies also reported subpopulation-specific loci associated with quantitative and qualitative traits in rice, which were veiled by the confounding effect in a GWA model combining all subpopulations [17,18].

Following previous recommendation, we used a combination of mixed model and more basic GLM for association discovery [18]. The GLM detected eighteen and four loci above the significance thresholds −log10(*p*) = 5 and 6, respectively. On the other hand, the MLM, known to perform a much stricter correction for population structure, detected three and one loci above two significance thresholds, respectively. Although the −log10(*p*) in the quantile-quantile (Q–Q) plot of the GLM was far from the expected distribution, thus with under-correction (Figure 5B), the top three loci on chromosomes 1 and 3 from the GLM were also detected in the MLM with reduced significance values (Figure 5A). It was therefore speculated that some loci detected in the GLM, especially a consistent major peak on chromosome 4 above the significance threshold −log10(*p*) = 6, were not false positives (over-estimation) but were possibly removed in the MLM due to over-correction.

For haplotype analysis to determine allelic effects on PHS resistance, we focused on four loci: three loci on chromosome 1 and three detected in both GLM and MLM and one locus on chromosome 4 detected in the GLM. Clear allelic effects were observed in two consistent loci on chromosome 1 and 4 (Figure 5A,C). In six accessions (genotype 1), the favorable haplotypes (shaded green) were present at both loci and this group showed a strong enhancement of PHS resistance, at 120% relative to genotype 5 having unfavorable haplotypes at both loci. Genotypes 2, 3, and 4, with only one favorable haplotype showed moderate enhancements, by 46~57%. However, there was no allelic effect at the other two loci on chromosome 1 and 3 (Figure 5A). A few accessions had minor haplotypes at these loci, but their PHS resistance was extremely low with averages of 20.46% and 29.98% at chromosome 1- and 3-loci, respectively (data not shown). Given that fact, these two loci were found to be statistically associated with the trait but not very useful to enhance PHS resistance.

## 3. Discussion

In many accessions, PHS resistance largely reduced between 20 and 30 DAF. This may indicate that the low number of germinated seed at 20 DAF was due to lack of germination promoters such as ABA regulators in immature seeds [19] rather than PHS resistance. Greenish color of seed coats frequently observed in the 20 DAF group was possibly evidence of seed immaturity. Because the timing of flowering and harvest maturity depends on climate conditions [20,21,22], optimal sampling timing for PHS screening should be individually determined in each cultivation region. For example, in the Republic of Korea under temperate climate with relatively low light intensity, and thus less efficient grain filling process than that of tropical climate [23], 42 DAF was the best sampling time for PHS screening [24]. It is reasonable to expect a large variation in PHS between rice ecotypes, since in addition to natural variation in dormancy that would be expected of wild progenitor populations adapting to different environments in course of their evolution, the impact of domestication on PHS in indica and japonica would have been different as well, since the extent of artificial selective pressure differed greatly between these groups, with japonica undergoing a much more severe bottleneck [25,26]. From the perspective of phylogeography, indica and japonica sub-groups are believed to have originated from highly diverged gene pools within wild progenitor species in the south of the Himalaya Mountain range and southern China, respectively [26,27], which explains large genetic distance between indica and japonica groups, whereas two japonica groups have diverged more recently and are relatively closer to each other. The six japonica accessions having over 90% of PHS resistance could be used in breeding programs to improve elite japonica varieties (Figure 2B and Figure 4), which may bring some advantages such as increase in spikelet fertility rate of mapping population progenies. In breeding of temperate japonica rice, indica donors often caused high sterility rates, hence additional efforts have been required to recover yield [28,29]. The mean phenotype in Group 1 (Figure 5C) was higher than what would be expected under the additive model, but this deviation from additivity was not statistically significant since Group 1 only contains six varieties. Although phenotype distribution among haplotype groups suggest a possibility of epistasis, this hypothesis requires future testing.

Based on the genome sequence database, a total of 41 genes comprised of 12 annotated, 13 expressed, 13 (retro)transposon and 3 hypothetical genes were located within identified loci on chromosome 1 (180 kb) and 4 (151 kb) [30]. Among annotated genes, 10 genes were expressed during seed maturity stages in previous transcriptome analyses (Table 1) [31]. Network analysis is a powerful tool to query potential interactions among genes that is supported by multiple lines of evidence including functional annotations, i.e., gene ontology (GO), co-expression, and other evidence [32]. We interrogated RiceNet v2 [33] with the candidate genes, and identified four that are potentially relevant to major PHS resistance mechanisms previously characterized in barley, wheat, and rice, such as its regulation by plant hormones [2,34]. Networks of LOC_Os01g03740 (annotated as nuclease PA3), LOC_Os01g03820 (C2 domain containing protein), LOC_Os01g03914 (cation efflux family protein), and LOC_Os04g08570 (uncharacterized PE-PGRS family protein) were found to involve regulation of ABA signaling pathway, and thus may affect dormancy/germination processes. We also interrogated the Rice Combined mutual Ranked Network (RCRN), which has been shown to allow detection of gene networks involving cell wall metabolisms that were experimentally validated [32]. The RCRN is an extension to RiceNet v2 [33] incorporating more data and two other networks. Interestingly, networks from both PA3 genes and the C2 domain protein, as well as LOC_Os01g03840 (annotated as C2H2 zinc finger protein), connected to loci with high edge strengths that were in common to loci identified with RiceNet v2 [33]. Further, for each of the genes, the gene ontology support between loci identified from either network were in general agreement.

In the case of wheat, *TaPHS1* (aka *TaMFT1*, a Mother of FT and TFL1 gene), cloned from a major PHS loci on chromosome 3AS, positively regulated ABA responses and suppressed germination [35,36]. LOC_Os03g16170 (annotated as protein phosphatase 2C), a network gene of LOC_Os01g03914 is known to upregulate gibberellic acid (GA), which has an antagonistic relationship with ABA [37]. Therefore, this gene may release seed from dormancy resulting in increase of PHS susceptibility. It has been reported that the seed dormancy locus-*qSD1-2* derived from indica rice involved in GA biosynthesis and its loss-of-function allele increased ABA concentration and consequently, PHS resistance [33]. Similarly, the Arabidopsis *AtMFT* gene functioned as a negative regulator of ABA signaling during germination [38]. Networks of LOC_Os01g03840 (annotated as ZOS1-02-C2H2 zinc finger protein) and LOC_Os01g03950 (glycosyl hydrolase, family 31) regulate auxin signaling pathway. Although ABA-auxin interactions vary in a tissue-dependent fashion [39], they work together to suppress seed germination; auxin enhances the inhibitory effects of ABA [40].

The flavonoid biosynthetic genes in rice (*R_C_*) and wheat (*R*_−1_) are known to strongly confer pleiotropic effects on seed coat color and dormancy via ABA signaling pathway [11,12]. However, in our GWA analysis, the association between PHS resistance and SNP markers located in the *R_C_* locus on chromosome 7 was not very significant as compared to that of the major loci on chromosomes 1 and 4 (Figure 5A). In the japonica panel used in this study, there were only seven accessions having red pericarp color and their PHS resistances were not very high with a mean of 50.12% (data not shown). This implies that the effect of favorable haplotypes on the chromosome 1 and 4 loci (Figure 5C) does not require a functional *R_C_* allele. From the perspective of breeding, it is difficult to use the *R_C_* locus as it dominantly confers the pigmentation in the mapping population [41]. Hence, we aimed to identify new PHS loci not associated with seed pericarp color. Use of the *Sdr4* locus was a successful story in terms of the breeding efficiency. It has been reported that a near isogenic line (NIL) carrying the *Sdr4* locus on chromosome 7 derived from aus variety Kasalath enhanced both PHS resistance and grain quality in Koshihikari, one of the most popular temperate japonica varieties [42]. A consistent peak at this locus was detected in our analysis but its significance value was lower than that of the major loci on chromosomes 1 and 4 (Figure 5A).

**Figure 6:**
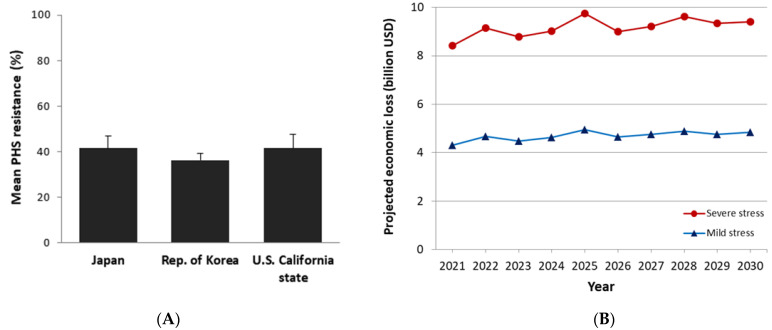
Projected economic loss by pre-harvest sprouting (PHS) damages on temperate japonica rice production in California state of the U.S., Japan, and the Republic of Korea: (**A**) Mean PHS resistance (%) of 48 temperate japonica varieties from those three countries used in this study; and (**B**) Projected economic loss by PHS between 2021 and 2030. The level of severe stress is the same as PHS screening conditions used in this study, whereas mild stress level is half of severe stress.

Climate change impact, i.e., new types of abiotic stresses induced by unexpected high temperature or flooding events, can cause a significant loss of crop harvest, which demands fast responses and solutions from crop research institutes. As we demonstrated in this study, screening diverse crop germplasm held at genebanks could be one of the most effective ways to swiftly find new donors to be used in breeding programs or direct seed distribution to farmers. Although many japonica accessions originating from various regions were susceptible to PHS, we expect that most countries of origin will have alternative ways to secure their food security. For example, South-Eastern Asian countries can grow indica varieties having high PHS resistance instead of japonica varieties. However, in Eastern Asian countries, such as Japan and the Republic of Korea, under temperate climate, it is not easy to widely grow indica or other rice sub-groups except temperate japonica since they are not adapted. The state of California in the USA is one of the top rice production areas and temperate japonica varieties are mainly grown due to their excellent grain quality as well as cool temperature during the rice planting season. Based on 10 years projection, the economic loss by PHS under severe and mild stresses could range between 8–10 billion USD and 4–5 billion USD, respectively, for those three countries combined (Figure 6B). Therefore, we plan to improve elite temperate japonica varieties for PHS resistance using the promising germplasm and candidate loci identified in this study.

## 4. Materials and Methods

### 4.1. Plant Materials

A diverse rice panel of 277 accessions from the 3000 Rice Genomes Project [43], representing temperate japonica (156 accessions), tropical japonica (97 accessions), and indica (24 accessions) sub-groups were grown on the Zeigler Experiment Station of the International Rice Research Institute (IRRI), Philippines, in the 2019 dry season. These accessions originated from 14 regions (60 countries) in Africa, Asia, Europe, North America, Oceania, and South America (summarized in Table 2, see Supplementary Material S2 for entry list) and seeds of the panel had been genetically purified through single-descendent harvest. Indica accessions were chosen as control based on their strong dormancy behavior in our previous study [44]. Twenty day-old seedlings were transplanted to the field, with 200 mm spacing of plants within and between rows. NPK (14-14-14) was applied at 28 kg ha^−1^ as basal dose and additional nitrogen (20 kg ha^−1^) was applied at 30 and 50 days after transplanting. Insect, disease, and weed control were as per standard procedures at IRRI.

### 4.2. PHS Screening

Three panicle samples per plant of each accession were harvested at 20 and 30 days after flowering (DAF). To induce the similar conditions to heavy rainfall during harvest period, after counting total seed number, samples were immediately rolled up in wet paper towel and incubated at 100% RH, 30 °C with 12 h light per day in a growth chamber. Seed germination was scored at 5, 10, and 15 d of incubation. PHS resistance value was calculated as a percentage of ungerminated seeds during incubation period.

### 4.3. GWA Analysis

Genome-wide association (GWA) analysis was performed using genome-wide efficient mixed model analysis software (GEMMA v. 0.95 alpha) [45]. The 4.8 million single nucleotide polymorphisms (SNP) marker set downloaded from the Rice SNP-Seek database (http://snp-seek.irri.org; accessed on 3 May 2021) [46] was filtered for 12% missing data and minor allele frequencies <5% using PLINK v. 1.9 [47,48] After removing highly correlated SNPs via LD (linkage disequilibrium) pruning with r^2^ = 0.99 using PLINK command “--indep-pairwise 20 kb 1 0.99”, the final data set-296 K SNPs were used for analysis. The kinship matrix and principal components (PC) were computed using GEMMA command “-gk 1” and PLINK “--pca”, respectively. The general linear model (GLM) with PC and mixed linear model (MLM) with kinship matrix and PC as fixed effect covariates were performed. Significance thresholds were calculated accordingly [49,50]. The estimates of the effective number of independent tests were 30,851 [49] and 37,156 [50], which correspond to cutoffs of −log10(*p*) for significance level alpha = 0.05 at 5.79 and 5.87, respectively. Based on this, the genome-wide significance level was set to the suggestive and conservative thresholds: −log10(*p*) = 5 and 6, respectively. SNPs in the region of identified loci were downloaded from the Rice SNP-Seek Database [51] and allelic effects on the phenotype were determined.

### 4.4. Projected Economic Loss

The projected economic losses associated with PHS in California, Japan, and the Republic of Korea, where PHS-prone temperate japonica rice are mainly grown, were calculated using data on projected yield, rice area, and farmgate price. We considered a simple revenue calculation which accounts for PHS damage on crop yield and the price discount due to grain quality damage. This projected yield data were generated based on stochastic deviation from historical yield trend in those three target countries based on historical yield amplitude. The projection was conducted using ORYZA crop simulation model [52]. The expected yield and farmgate prices are likely to be affected by the severity of stress conditions. We therefore considered two scenarios: severe stress whose level is the same as PHS screening conditions used in this study (Figure 6A) and mild stress, a half-level of severe stress. Price discount due to grain quality damage was applied in the calculation as following the emergency market price adjustment for damaged rice, which was made by the Ministry of Agriculture, Food, and Rural Affairs (MAFRA), the Republic of Korea [53], right after a big typhoon event and consequent PHS damages on rice production in October 2019:

- 76.9% of original price when portion of damaged grains is less than 25%;
- 64.1% of original price when portion of damaged grains is less than 35%; and
- 51.3% of original price when portion of damaged grains is less than 50%.

## Figures and Tables

**Table 1:** List of a priori candidate genes enhancing pre-harvest sprouting tolerance in japonica rice. For the RCRN, a not significant (N.S.) value is the maximum edge strength.

No	Locus ID	Position	Expression during Seed Development ^1^	Annotation	Network Gene	Gene Ontology
					RiceNet v2	RiceNet v2
					RCRN	RCRN
1	LOC_Os01g03740	1566554–1562612	Seed-5 DAP ^2^	Nuclease PA3	LOC_Os05g31750/ LOC_Os02g36390/ LOC_Os06g13040	Response to abscisic acid stimulus and abiotic stresses/pollen exine formation/photoperiodism
					LOC_Os02g36390/ LOC_Os10g39640/ LOC_Os05g31750/	Sugar transporter/cell wall growth/response to abiotic stimulus
2	LOC_Os01g03730	1566138–1569472	Seed-5 DAP	Nuclease PA3	LOC_Os01g65440/ LOC_Os02g09220/ LOC_Os06g17830	Response to stress/oxidation reduction/DNA demethylation and repair
					LOC_Os01g65440/ LOC_Os08g15020/ LOC_Os01g46370	Response to stress/MYB transcription factor/lipid metabolism
3	LOC_Os01g03820	1597486–1604610	Embryo-25 DAP	C2 domain containing protein	LOC_Os08g02140/ LOC_Os06g46670/ LOC_Os01g07760	Protein ubiquitination/G-protein coupled receptor protein signaling pathway/positive regulation of seed germination and abscisic acid mediated signaling pathway
					LOC_Os08g08190/ LOC_Os01g53160/ LOC_Os08g02140	Unknown molecular function/plasma membrane/response to abiotic stimulus/protein modification
4	LOC_Os01g03840	1625159–1626771	Embryo-25 DAP	ZOS1-02–C2H2 zinc finger protein	LOC_Os08g41950/ LOC_Os05g38120/ LOC_Os01g18360	Flower development; specification of floral organ number/regulation of transcription, DNA-dependent/auxin mediated signaling pathway
					LOC_Os05g38120/ LOC_Os02g57790/ LOC_Os01g72020/	Cell differentiatio/post-embryonic development/DNA binding transcription factor/homeodomain protein/nucleic acid binding/flower development
5	LOC_Os01g03890	1657315–1658388	Seed-5 DAP	DUF260 domain containing protein	N/A	N/A
					N.S. (0.01)	
6	LOC_Os01g03914	1677129–1673010	Embryo-25 DAP	Cation efflux family protein	LOC_Os05g25310/ LOC_Os01g62760/ LOC_Os03g16170	Long-chain fatty acid metabolic process/negative regulation of abscisic acid mediated signaling pathway/positive regulation of seed germination and gibberellic acid mediated signaling pathway; release of seed from dormancy
					LOC_Os10g02210/ LOC_Os06g21820/ LOC_Os07g23944	Peptide transporter/protein binding/multicellular development/carbohydrate hydrolase
7	LOC_Os01g03950	1698203–1703090	Seed-5 DAP	Glycosyl hydrolase, family 31	LOC_Os07g43390/ LOC_Os06g09630/ LOC_Os04g56070	Maltose, carbohydrate, starch and glucose metabolic processes/fatty acid biosynthetic process; embryonic development/response to auxin stimulus
					LOC_Os01g67220/ LOC_Os01g07120/ LOC_Os06g34690	Carbohydrate hydrolase/response to abiotic stress stimulus/DNA binding transcription factor/
8	LOC_Os04g08460	4557188–4559281	Seed-5 DAP	OsFBX115–F-box domain containing protein	N/A	N/A
					N.S. (0.05)	
9	LOC_Os04g08470	4571423–4564403	Seed-5 DAP	OsFBX116–F-box domain containing protein	N/A	N/A
					N.S. (0.01)	
10	LOC_Os04g08570	4652730–4651469	Seed-10 DAP	Uncharacterized PE-PGRS family protein	LOC_Os01g01350/ LOC_Os03g04360/ LOC_Os10g01560	Protein transport/response to abscisic acid stimulus/abscisic acid mediated signaling pathway; seed germination
					LOC_Os07g13830/ LOC_Os12g30500/ LOC_Os03g31430	Putative protein ubiquination/protein binding/lipid metabolic process

^1^ Based on previous transcriptome analysis (MSU v7 annotation, http://rice.uga.edu/; accessed on 20 April 2021) [30]). ^2^ Days after pollination.

**Table 2:** The diverse rice panel used in this study.

Continent	Region	No. of Accession	Ecotype		
			Indica	Temperate Japonica	Tropical Japonica
Africa	Eastern Africa	7	3	0	4
	Western Africa	7	2	0	5
	Northern Africa	16	2	2	12
Asia	Eastern Asia	71	0	66	5
	South-Eastern Asia	54	6	6	42
	Southern Asia	27	8	8	11
Europe	Eastern Europe	9	0	9	0
	Central Europe	7	0	7	0
	Southern Europe	36	0	35	1
	Western Europe	7	1	4	2
	Northern Europe	1	0	1	0
North America	North America	13	1	8	4
Oceania	Oceania	4	0	4	0
South America	South America	18	1	6	11
Total		277	24	156	97

## Data Availability

The data generated in this study are available from the corresponding author upon reasonable request.

## Supplementary Materials

The following are available online at https://www.mdpi.com/article/10.3390/plants10081709/s1, Supplementary Material S1. Genome-wide association analysis of pre-harvest sprouting (PHS) in the complete dataset (24 Indica and 253 Japonica rice accessions), Supplementary Material S2: The list of 277 rice accessions used in this study.
