# Supplementary material for: Novel Sources of Pre-Harvest Sprouting Resistance for Japonica Rice Improvement
Source: Plants (Basel). 2021 Aug 19;10(8):1709. doi: 10.3390/plants10081709 (PMC8401653; doi:10.3390/plants10081709)
Supplement: Supplementary file 1 [file plants-10-01709-s001.zip › Supplementary Material S1.pdf]

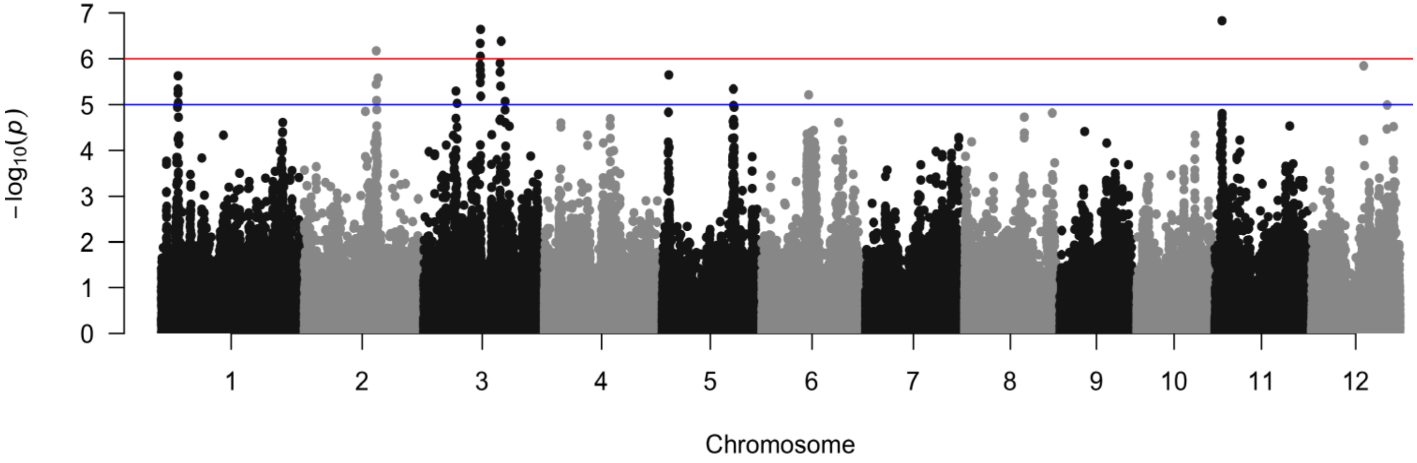

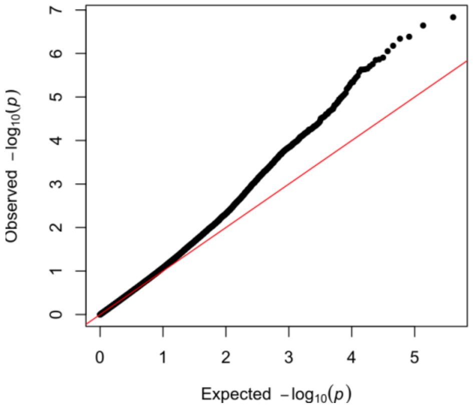

Supplementary Material S1. Genome-wide association analysis of pre-harvest sprouting (PHS) in the complete dataset (24 Indica and 253 Japonica rice accessions). Manhattan and Quantile-quantile plots were generated through the mixed linear model (MLM) with kinship matrix and principal components. Points above the blue and red threshold lines indicate significant association at -log10(*p*)=5 and 6, respectively.
